# Global and regional quality of care index (QCI) by gender and age in oesophageal cancer: A systematic analysis of the Global Burden of Disease Study 1990–2019

**DOI:** 10.1371/journal.pone.0292348

**Published:** 2023-10-03

**Authors:** Shabnam Iezadi, Narges Ebrahimi, Seyyed-Hadi Ghamari, Zahra Esfahani, Negar Rezaei, Erfan Ghasemi, Sahar Saeedi Moghaddam, Sina Azadnajafabad, Zhaleh Abdi, Zahra Shokri Varniab, Ali Golestani, Ashkan Pourabhari Langroudi, Arezou Dilmaghani-Marand, Yosef Farzi, Hamid Pourasghari

**Affiliations:** 1 Research Center for Emergency and Disaster Resilience, Red Crescent Society of the Islamic Republic of Iran, Tehran, Iran; 2 Hospital Management Research Center, Health Management Research Institute, Iran University of Medical Sciences, Tehran, Iran; 3 Non-Communicable Diseases Research Center, Endocrinology and Metabolism Population Sciences Institute, Tehran University of Medical Sciences, Tehran, Iran; 4 Department of Biostatistics, University of Social Welfare and Rehabilitation Sciences, Tehran, Iran; 5 Kiel Institute for the World Economy, Kiel, Germany; 6 National Institute of Health Research (NIHR), Tehran University of Medical Sciences (TUMS), Tehran, Iran; 7 Endocrinology and Metabolism Research Center, Endocrinology and Metabolism Clinical Sciences Institute, Tehran University of Medical Sciences, Tehran, Iran; 8 School of Health Management and Information Sciences, Iran University of Medical Sciences, Tehran, Iran; Tehran University of Medical Sciences, ISLAMIC REPUBLIC OF IRAN

## Abstract

**Background:**

The aim of this study was to examine the quality of care by age and gender in oesophageal cancer using Global Burden of Disease (GBD) database.

**Methods:**

Patients aged 20 and over with oesophageal cancer were included in this longitudinal study using GBD 1990–2019 data. We used the Socio-Demographic Index (SDI) to classify the regions. We used Principal Component Analysis (PCA) method to calculate the Quality of Care Index (QCI). The QCI was rescaled into a 0–100 single index, demonstrating that the higher the score, the better the QC.

**Results:**

The age-standardized QCI for oesophageal cancer dramatically increased from 23.5 in 1990 to 41.1 in 2019 for both sexes, globally. The high SDI regions showed higher QCI than the rest of the regions (45.1 in 1990 and 65.7 in 2019) whereas the low SDI regions had the lowest QCI, which showed a 4.5% decrease through the years (from 13.3 in 1990 to 12.7 in 2019). Globally, in 2019, the QCI showed the highest scores for patients aged 80–84, reported 48.2, and the lowest score for patients aged 25–29 reported 31.5, for both sexes. Globally, in 2019, age-standardized Gender Disparity Ratio (GDR) was 1.2, showing higher QCI in females than males.

**Conclusion:**

There were fundamental differences in the QCI both globally and regionally between different age groups as well as between males and females. To achieve the goal of providing high-quality services equally to people in need in all over the world, health systems need to invest in effective diagnostic services, treatments, facilities, and equipment and to plan for screening and surveillance of high-risk individuals.

## Introduction

Oesophageal cancer is the seventh most prevalent cancer of all types and the sixth leading cause of cancer mortality, irrespective of sex and age, worldwide, accounting for 604,100 new cases in 2020 and causing around one-third of all disability-adjusted life-years (DALYs) due to cancer [1]. Many risk factors including sex, age, genetics, lifestyle, and geographical and environmental factors contribute to oesophageal cancer incidence [2,3]. For example, oesophageal cancer is around two to three times more prevalent among males than females [1,3], and it is more frequent in older age [2]. The differences in the incidence of oesophageal cancer among people of different ages, sex, and socioeconomic status, along with the increasing growth of the disease [2,3], raise the concern regarding the Quality of Care (QC) and age and gender disparities in oesophageal cancer.

Improvement of the QC, which can contribute to an increase in survival-year and quality of life in patients with cancer, is one of the most fundamental challenges that health systems worldwide face [4]. QC has been proven to differ broadly among countries and regions, which calls on researchers to conduct more research in this field [5,6]. The evaluation methods of QC are incongruous, and researchers have considered different measures and factors to evaluate the different aspects of the QC in healthcare settings [7]. For instance, some researchers have considered structure, process, and outcome to assess the QC [8]. Some other studies have focused on patient satisfaction [9], patients’ expectations and perceptions [10,11], and patients’ outcomes [12,13]. In addition, some studies have adopted a more specialized approach, namely technical quality, focusing on adherence to the standard protocols of care [14]. The common aim of all approaches mentioned above to assessing the QC is the improvement of the health outcomes; therefore, using a composite metric that encompasses outcome measures, such as DALYs, Mortality-to-Incidence Ratio (MIR), Years of Life Lost (YLLs), and Years Lived with Disability (YLDs), have proven to be an appropriate measure to evaluate the QC in cancer studies [5,6].

In addition to differences in QC for patients with oesophageal cancer between countries, notable disparities are attributed to oesophageal cancer across social groups within populations [15]. For example, one type of oesophageal cancer, oesophageal adenocarcinoma, shows a striking disparity between males and females, which varies across different countries [16]. Moreover, there is evidence that the incidence risk of the other type of oesophageal cancer and oesophageal squamous cell carcinoma is associated with lower socioeconomic status [17,18]. Oesophageal cancer has even affected high-income countries and low- and middle-income countries (LMICs) unequally [19]. For instance, a global study on oesophageal cancer showed that countries with a low sociodemographic index (SDI) had disproportionately higher oesophageal squamous cell carcinoma cases [19]. Exploring disparities in oesophageal cancer care is a prerequisite to correcting them through developing effective interventions to alleviate disparities and linking disparities to quality-of-care measure [20,21].

There is no doubt that analyzing the global and regional data to understand the QC and disparities in the context of oesophageal cancer is of principal value. Such studies can contribute to a better understanding of the differences in the quality of care and disparities among regions with different levels of income and different sociodemographic statuses, resulting in the identification of regions affected more severely by inequalities in healthcare [22]. The Global Burden of Disease (GBD) provides a tool to quantify health outcomes and help health systems and policymakers to understand the real characteristic of their country’s health challenges and improve any kind of disparities in healthcare [23]. Incorporating both “the prevalence of a given disease or risk factor and the relative harm it causes”, GBD goes beyond just estimating disease prevalence. GBD allows the comparison of different health outcomes in different countries and territories. Moreover, the GBD’s machinery design contribute to regular and timely updates when new data and epidemiological studies are approached. A study by GBD Oesophageal Cancer Collaborators in 2017 examined the burden of disease regionally and globally; however [24], the present study has added the Quality of Care Index (QCI) as a composite measure to demonstrate the quality of care and its variations in regions with different SDI, by using the next generation of GBD data. In this study, we aimed to examine the QC and gender and age disparities in oesophageal cancer using the GBD database updated in 2019.

## Methods

The GBD 2019 study provides a comprehensive synthesis of the available evidence on global, regional, and national-level fatal and non-fatal health outcomes, disease risk factors and injury causes, and health system measures. GBD 2019 coveres 204 countries and territories from 1990 to 2019.[25] In this study, using GBD 1990–2019 data [23], we globally and regionally examined the QC with gender and age disparities in QC for patients aged 20 and over with oesophageal cancer. We classified the regions by using SDI, which was described as the average of per capita income, education, and fertility of world regions to provide the reports by regions [26]. So that we categorized regions as low SDI, lower-middle SDI, middle SDI, higher-middle SDI, and high SDI. We also used time trends to show the QC and gender disparities over time from 1990 to 2019.

We used WHO’s 10^th^ revision of the International Classification of Diseases (ICD-10) system to define the disease under diagnosis codes of C15-C15.9 and Z85.01 to define new cases and C15-C15.9, D00.1 D13.0 to map deaths [27,28].

### Quality of Care (QC)

QCI was used to evaluate the QC. We used Principal Component Analysis (PCA) method to calculate the QCI score [29]. PCA is a unique approach to factor analysis that converts the primary variables into a smaller set of linear compositions by considering the total variance in the data. That is, PCA transforms a set of intercorrelated variables into fewer dimensions, called components, with large size of the variability of the original variables. The components can unite highly correlated variables within each component while being uncorrelated one to another [30]. The first principal component, which is a linear combination of all variables, was considered QCI.

In the first place, we acquired age-standardized primary measures from GBD 2019 to calculate four subsequent secondary measures [23]. Afterward, we used four secondary measures, including MIR, DALYs-to-Prevalence ratio, YLL-to-YLD ratio, and Prevalence-to-Incidence ratio, to composite them into a single measure named QCI. The higher the rates of the measures the lower QC were. The four measures were formulated as:

$$MIR=\frac{Death(x)}{Incidence(x)}$$

$$DALYs\;to\;Prevalence\;(x)=\frac{DALYs(x)}{Prevalence(x)}$$

$$Prevalence\;to\;Incidence\;(x)=\frac{Prevalence(x)}{Incidence(x)}$$

$$YLL\;to\;YLD\;(x)=\frac{YLL(x)}{YLD(x)}$$

Where x is the region classified either based on SDI classification of regions.

PCA score for each region was used to combine the four above-mentioned secondary measures. The QCI, derived from the PCA score, was rescaled into a 0–100 single index, demonstrating that the higher the score, the better the QC. The QCI formulated as:

$$QCI\;(x)=\frac{\left[PCAscore(x)-minPCAscore\right]}{\left[maxPCAscore-minPCAscore\right]}$$

### Disparities in the QC

We assessed disparities in the QC in oesophageal cancer-targeting two main criteria: age disparity and gender disparity. In order to examine age disparities, we calculated QCI for each age group classifying the ages as five years intervals, starting from 20 years old up to 85 years old and above.

In order to examine gender disparity, we used Gender Disparity Ratio (GDR) formulated as the proportion of QCI for females to QCI for males in a region. According to this formula, values closer to 1 show the lower gender disparity. Values greater than one show higher QCI in female patients compared to males and vice versa.

### Statistical analysis

We calculated age-standardized values per 100,000 population for the primary indices reporting 95% uncertainty interval (UI). Lack of overlap in UI’s of comparison groups was the criterion to consider that the estimations and shifting trends were significant. We used R statistical packages v3.4.3 (http://www.rproject.org/, RRID: SCR_001905) to perform statistical analyses and visualizations.

### QCI validity analysis

We used a mixed-effect model considering QCI as a dependent variable and inpatient admissions per capita, outpatient visits per capita, attributed age-standardized rate to all risk factors of esophageal cancer, the age-standardized death rate due to esophageal cancer, and age-standardized prevalence rate due to esophageal cancer as independent variables by random effects of countries. In order to examine the validity of QCI, we estimated the correlation between the predicted QCI and Healthcare Access and Quality Index (HAQI) and Universal Health Coverage (UHC) effective coverage index. Whereas HAQI was already introduced by Institute for Health Metrics and Evaluation (IHME) as a proxy of quality and access [31], UHC adequate coverage index was assessed by GBD 2019 Universal Health Coverage Collaborators for 204 countries and territories from 1990 to 2019 using measurement framework developed through WHO’s Thirteenth General Programme of Work (GPW13) [32]. In order to allow comparison, we adopted age-standardized values. The index’s correlation with the UHC effective coverage index and HAQI were approved by Pearson correlation coefficient of 0.62 and 0.59, respectively.

## Results

Globally, the age-standardized incidence rate of oesophageal cancer per 100,000 population in 2019 was 6.5 (95% UI, 5.7 to 7.2), and its age-standardized prevalence rate was 11.6 (95% UI, 10.1 to 12.9). All variables including numbers of DALYs, death, incidence, prevalence, YLDs, and YLLs showed an increase since 1990, globally. Nevertheless, the age-standardized rate per 100,000 demonstrated a decrease for all variables, globally (Table 1). Among the regions stratified based on SDI, those with higher-middle SDI and middle SDI had the higher age-standardized rate of incidence [7.1 (95% UI, 5.5 to 8.2) per 100,000 population] and prevalence rate [14 (95% UI, 11.1 to 16.3) per 100,000 population], respectively. In contrast, those with lower-middle SDI demonstrated the lowest age-standardized rates for incidence and prevalence reported 4.3 (95% UI, 3.9 to 6.2) and 6.2 (95% UI, 5.5 to 8.8), respectively. Regions with high SDI showed the lowest decrease in the number of oesophageal cancer deaths since 1990, reported 8.9 (95% UI, -12.3 to -5.1) decrease, while those with middle SDI by 42.3 (95% UI, -52.2 to -17.2) decrease had the highest decrease in the number of deaths (Table 1).

**Table 1:** Descriptive information on DALYs, deaths, incidence, prevalence, YLDs, and YLLs for oesophageal cancer, globally and regionally (1990–2019).

Location	Year	All ages (number)						Age-standardized (rate per 100,000)					
		DALYs	Deaths	Incidence	Prevalence	YLDs	YLLs	DALYs	Deaths	Incidence	Prevalence	YLDs	YLLs
Global	1990	8,208,267 (6,334,289 to 9,075,711)	319,332 (248,666 to 350,802)	319,969 (253,395 to 351,210)	489,194 (388,238 to 536,586)	85,089 (58,586 to 112,017)	8,123,178 (6,272,962 to 8,977,846)	199.3 (154.2 to 220)	8.2 (6.4 to 9)	8.1 (6.4 to 8.8)	12 (9.6 to 13.1)	2.1 (1.5 to 2.8)	197.2 (152.7 to 217.7)
	2019	11,666,017 (10,378,747 to 12,938,949)	498,067 (438,411 to 551,462)	534,563 (466,513 to 595,342)	960,610 (840,399 to 1,068,485)	150,074 (107,070 to 195,662)	11,515,943 (10,243,446 to 12,787,773)	139.8 (124.4 to 155)	6.1 (5.4 to 6.8)	6.5 (5.7 to 7.2)	11.6 (10.1 to 12.9)	1.8 (1.3 to 2.4)	138 (122.8 to 153.1)
	1990 to 2019 (%)	42.1 (23.1 to 75.5)	56 (36.6 to 88.2)	67.1 (46.5 to 98.5)	96.4 (72.6 to 131.9)	76.4 (53.7 to 108.9)	41.8 (22.6 to 75.2)	-29.9 (-39.2 to -14)	-25.3 (-34.5 to -10.3)	-19.3 (-29 to -4.4)	-3.6 (-15.2 to 13.5)	-14.2 (-24.9 to 1.1)	-30 (-39.4 to -14.2)
High SDI	1990	1,116,159 (1,091,372 to 1,135,517)	47,439 (45,967 to 48,389)	52,157 (50,608 to 53,200)	96,681 (93,981 to 98,748)	15,058 (10,926 to 19,312)	1,101,101 (1,076,725 to 1,120,168)	111.3 (108.9 to 113.2)	4.6 (4.4 to 4.7)	5.1 (4.9 to 5.2)	9.5 (9.3 to 9.8)	1.5 (1.1 to 1.9)	109.8 (107.5 to 111.7)
	2019	1,653,972 (1,570,861 to 1,731,345)	79,088 (73,600 to 83,089)	95,911 (86,719 to 105,092)	219,163 (197,989 to 241,176)	29,623 (21,314 to 38,245)	1,624,350 (1,542,691 to 1,699,854)	95.8 (91.4 to 100.5)	4.2 (3.9 to 4.4)	5.2 (4.7 to 5.7)	12.3 (11.1 to 13.6)	1.6 (1.2 to 2.1)	94.2 (89.9 to 98.5)
	1990 to 2019 (%)	48.2 (42.4 to 54.8)	66.7 (59.6 to 73.6)	83.9 (68.2 to 100.9)	126.7 (105.6 to 149.8)	96.7 (78 to 117.1)	47.5 (41.8 to 54.1)	-13.9 (-17.2 to -9.9)	-8.9 (-12.3 to -5.1)	2.5 (-6.7 to 11.9)	29 (16.6 to 42.5)	10.9 (0.1 to 22.6)	-14.2 (-17.4 to -10.3)
High-middle SDI	1990	2,235,279 (1,851,568 to 2,479,332)	88,112 (73,501 to 96,912)	86,734 (73,335 to 95,038)	127,273 (106,634 to 140,142)	22,746 (16,159 to 29,921)	2,212,533 (1,828,800 to 2,453,294)	203 (168.2 to 224.7)	8.3 (7 to 9.1)	8.1 (6.9 to 8.8)	11.6 (9.7 to 12.8)	2.1 (1.5 to 2.8)	200.9 (166.1 to 222.5)
	2019	3,105,596 (2,487,365 to 3,596,286)	135,757 (108,339 to 156,606)	145,151 (113,067 to 169,189)	255,801 (195,116 to 300,313)	40,278 (27,243 to 54,660)	3,065,318 (2,451,806 to 3,550,219)	151 (121.2 to 174.7)	6.6 (5.3 to 7.6)	7.1 (5.5 to 8.2)	12.4 (9.5 to 14.5)	2 (1.3 to 2.7)	149.1 (119.4 to 172.6)
	1990 to 2019 (%)	38.9 (16.8 to 64.8)	54.1 (31.1 to 81.7)	67.4 (41.2 to 99.1)	101 (68.9 to 140.7)	77.1 (47.6 to 111.5)	38.5 (16 to 64.8)	-25.6 (-37.5 to -11.8)	-20.6 (-32.4 to -6.7)	-12.7 (-26.2 to 3.6)	7.1 (-9.9 to 28)	-6.8 (-22.4 to 10.9)	-25.8 (-37.8 to -11.9)
Middle SDI	1990	3,574,428 (2,120,546 to 4,144,035)	138,218 (80,643 to 159,517)	136,240 (81,440 to 157,389)	200,314 (118,186 to 232,065)	35,184 (19,814 to 47,777)	3,539,244 (2,099,113 to 4,099,535)	330.1 (194.3 to 382.9)	14.1 (8.4 to 16.2)	13.5 (8.1 to 15.5)	18.7 (11.1 to 21.7)	3.4 (1.9 to 4.6)	326.7 (192.2 to 378.5)
	2019	4,485,644 (3,737,169 to 5,192,821)	193,720 (157,830 to 223,774)	170,414 (142,732 to 194,526)	355,071 (282,700 to 414,306)	56,186 (38,520 to 75,152)	4,429,457 (3,685,983 to 5,144,017)	175.2 (145.4 to 202.5)	8.1 (6.5 to 9.4)	8.4 (6.7 to 9.7)	14 (11.1 to 16.3)	2.3 (1.5 to 3)	172.9 (143.7 to 200)
	1990 to 2019 (%)	25.5 (1.5 to 91.2)	40.2 (15 to 107.4)	25.1 (2.6 to 88.1)	77.3 (44.2 to 156.9)	59.7 (29.8 to 128.5)	25.2 (1.1 to 90.7)	-46.9 (-56.6 to -20.1)	-42.3 (-52.2 to -17.2)	-48 (-56.8 to -23.4)	-25.4 (-38.9 to 6.8)	-33.4 (-45.4 to -7)	-47.1 (-56.8 to -20.2)
Low-middle SDI	1990	863,275 (763,527 to 1,104,285)	30,842 (27,388 to 39,603)	30,394 (26,887 to 39,269)	44,174 (38,923 to 56,965)	8,231 (5,648 to 11,423)	855,044 (754,885 to 1,092,675)	132 (117 to 169)	5.3 (4,7 to 6.9)	5.1 (4.5 to 6.5)	6.9 (6.1 to 8.9)	1.3 (0.9 to 1.8)	130.6 (115.8 to 167.3)
	2019	1,611,655 (1,433,392 to 2,250,321)	60,670 (53,987 to 85,565)	59,434 (52,249 to 83,651)	89,058 (78,281 to 124,219)	16,369 (11,416 to 23,584)	1,595,286 (1,417,742 to 2,230,246)	111.3 (99 to 155.7)	4.5 (4 to 6.4)	4.3 (3.9 to 6.2)	6.2 (5.5 to 8.8)	1.2 (0.8 to 1.7)	110.1 (97.9 to 154.3)
	1990 to 2019 (%)	86.7 (60.5 to 116.8)	96.7 (70.2 to 127.3)	95.5 (67.5 to 126.1)	101.6 (72.1 to 133.8)	98.9 (69.7 to 131.3)	86.6 (60.5 to 116.5)	-15.7 (-27.2 to -2.4)	-15.2 (-26.5 to -2)	-14.4 (-26.3 to -1.4)	-9.3 (-22.2 to 4.8)	-12 (-24.7 to 2.3)	-15.7 (-27.3 to -2.6)
Low SDI	1990	417,176 (342,900 to 481,147)	14,642 (12,029 to 16,772)	14,366 (11,901 to 16,558)	20,636 (17,041 to 23,835)	3,849 (2,648 to 5,175)	413,326 (339,467 to 476,687)	159.6 (131.3 to 183.2)	6.4 (5.2 to 7.3)	6 (5 to 6.9)	8.1 (6.7 to 9.2)	1.6 (1.1 to 2.1)	158 (129.9 to 181.7)
	2019	805,543 (662,160 to 973,246)	28,684 (23,834 to 34,252)	25,861 (21,419 to 30,571)	41,265 (33,963 to 49,382)	7,575 (5,104 to 10,699)	797,969 (655,600 to 961,449)	141.1 (116.5 to 168.9)	5.7 (4.8 to 6.8)	5.4 (4.5 to 6.4)	7.4 (6.1 to 8.8)	1.4 (0.9 to 2)	139.7 (115.4 to 167.2)
	1990 to 2019 (%)	93.1 (64.1 to 131.3)	95.9 (68.9 to 129.4)	80 (55 to 110.8)	100 (68.6 to 138.7)	96.8 (66.5 to 133.6)	93.1 (64.1 to 131.2)	-11.6 (-24 to 4.5)	-10.9 (-22.3 to 3.5)	-17.6 (-28.2 to -4.3)	-8 (-21.4 to 8.3)	-9.9 (-22.6 to 6.2)	-11.6 (-24 to 4.5)

Data in parentheses are 95% Uncertainty Intervals (95% UIs); DALYs = Disability-Adjusted Life Years; YLDs = Years Lived with Disability; YLLs = Years of Life Lost.

### Quality of Care Index (QCI)

The age-standardized QCI score for oesophageal cancer dramatically increased from 23.5 in 1990 to 41.1 in 2019 for both sexes globally (Table 2). This increase was much more evident in females than in males, reportedly 90.9% (from 26.4 to 50.4) compared to 57.0% (from 26.0 to 40.8). High SDI regions had higher QCI score than the rest of the regions (45.1 in 1990 and 65.7 in 2019) whereas the low SDI regions had the lowest QCI score, which showed a 4.5% decrease through the years (from 13.3 in 1990 to 12.7 in 2019).

**Table 2:** Age-standardized QCI scores for oesophageal cancer, globally and regionally (1990–2019).

Population		QCI scores*	
		1990	2019
**Global**	Overall	23.5	41.1
	Female	26.4	50.4
	Male	26.0	40.8
**High SDI**	Overall	45.1	65.7
	Female	53.1	73.9
	Male	45.2	65.6
**High-middle SDI**	Overall	20.1	40.7
	Female	24.5	58.8
	Male	22.2	37.7
**Middle SDI**	Overall	18.5	32.6
	Female	22.2	52.0
	Male	20.7	27.3
**Low-middle SDI**	Overall	15.5	19.8
	Female	16.8	21.2
	Male	19.1	23.2
**Low SDI**	Overall	13.3	12.7
	Female	14.8	16.9
	Male	16.9	14.3

SDI = Socio-Demographic Index; QCI = Quality of Care Index; All scores are age-standardized.

* Scores 0–100; 0 = the worst quality of care, and 100 = the best quality of care.

While low SDI regions revealed a 4.6% decrease in the QCI score of oesophageal cancer from 1990 to 2019, higher-middle SDI, middle SDI, high SDI, and lower-middle SDI regions demonstrated increases in the QCI scores reported 102.9%, 76.8%, 45.7%, and 27.7%, respectively.

Females from all SDI classifications showed an increase in QCI score with the highest increase related to higher-middle SDI and middle SDI regions (approximately 139% increase) and the lowest increase related to low SDI regions (14.7% increase). Whereas males from low SDI regions had 14.9% decrease in the QCI score, those from the other SDI classification regions showed an increase in the QCI score with the highest increase from higher-middle SDI regions (69.6% increase) and lowest increase from lower-middle SDI regions (21.5% increase).

### Age disparities in the QC

QCI for oesophageal cancer in 2019 demonstrated variations in different age groups, globally. In 2019, the QCI showed the highest score for patients aged 80–84 reported 48.2, and the lowest score for patients aged 25–29 reported 31.5, for both sexes.

**Fig 1:**
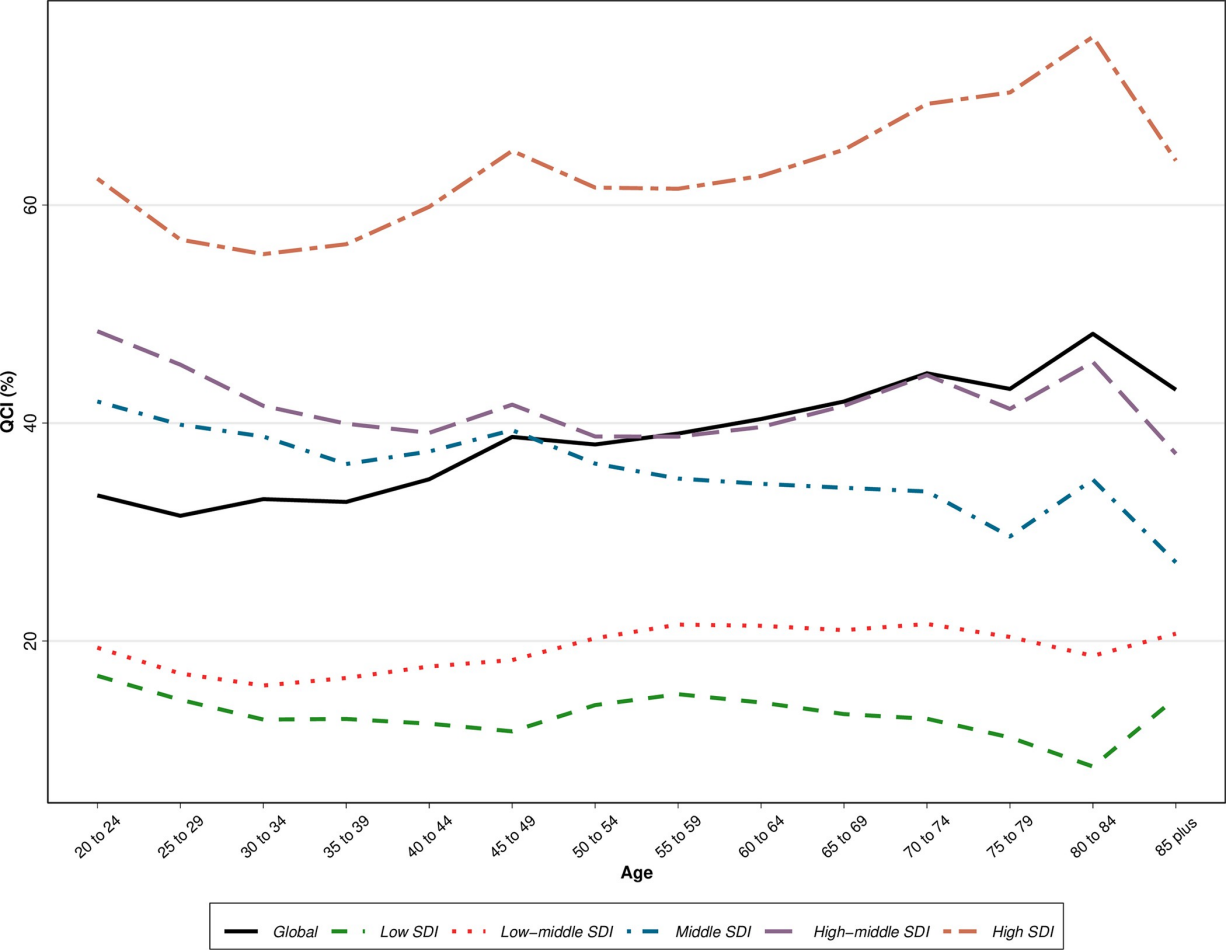
Age disparity patterns of quality of oesophageal cancer care globally and regionally based on sociodemographic index (SDI) quintile.

Considering the regions classified by SDI, patients aged 80–84 from high SDI regions showed the highest QCI score, and those from low SDI showed the lowest QCI score reported 75.4 and 8.5, respectively. In the middle SDI region, patients aged 75–89 with QCI score of 29.6, and in the higher-middle SDI region, patients aged 40–44 with QCI score of 39.1 showed the lowest QCI score in each region. In the high SDI region, patients of age groups 25–29, 30–34, and 35–39 showed the lowest QCI in that region scored 56.8, 55.5, and 56.4, respectively. Fig 1 shows the QCI score classified by age groups of five-year intervals globally and regionally by SDI classifications.

### Gender disparities in the QC

Globally, in 2019, age-standardized GDR score was higher than 1 (1.2), which shows the higher QCI score in females than males. This was also the case when categorizing age in two separate groups, including 20-50-year-old and above 50-year-old.

**Fig 2:**
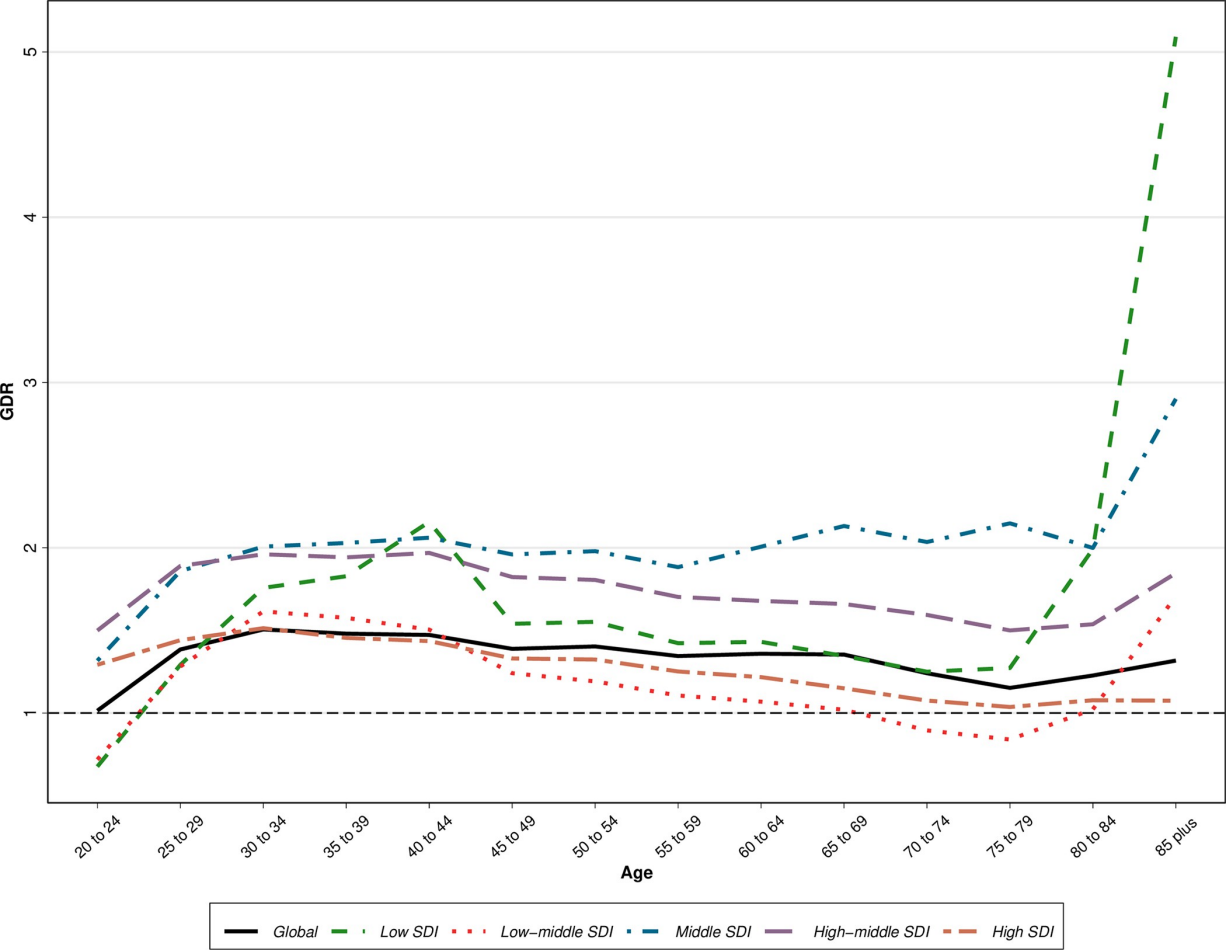
Gender disparity patterns of quality of oesophageal cancer care globally and regionally based on sociodemographic index (SDI) quintile.

**Fig 3:**
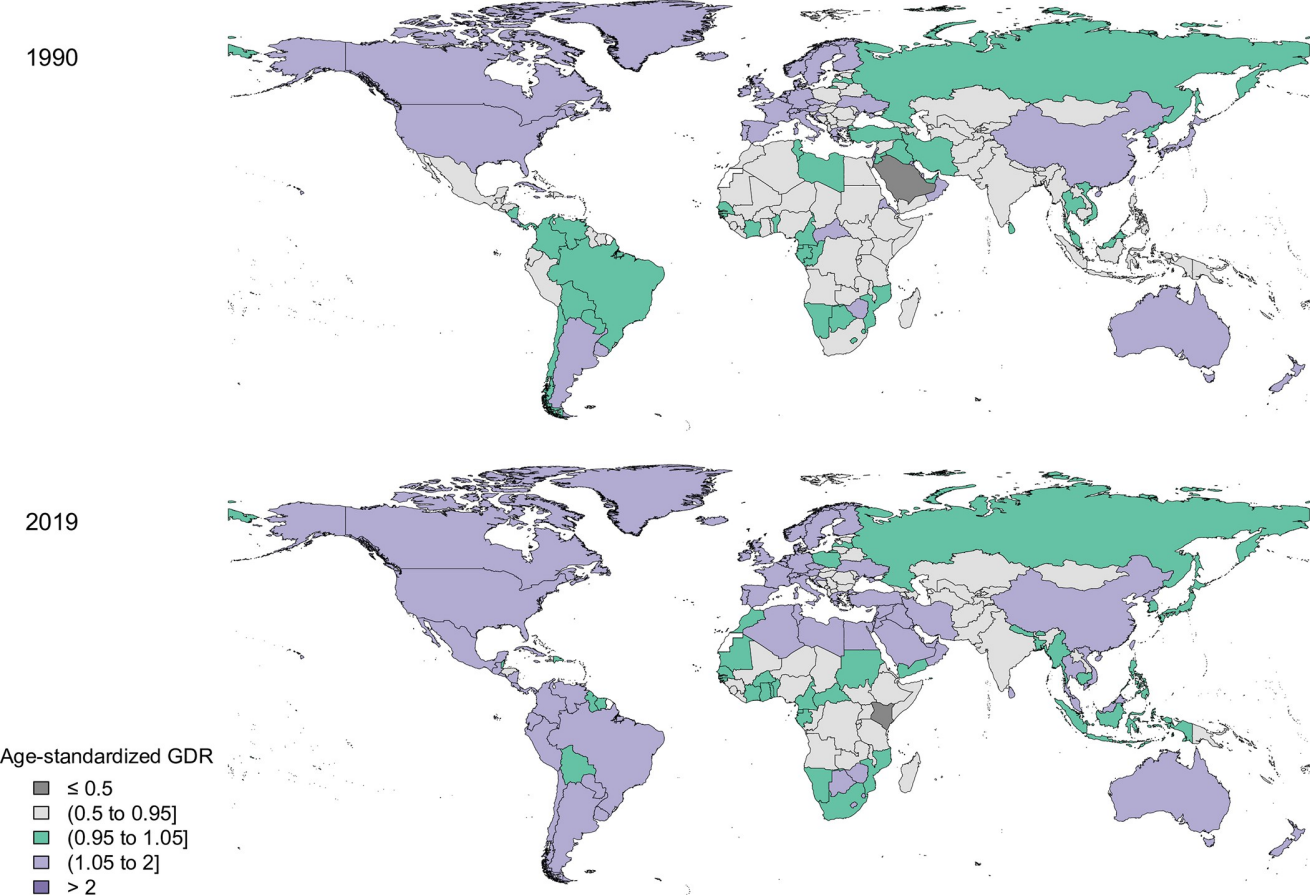
Age-standardized gender disparity ratio (GDR) map for Quality of Care Index (QCI) in oesophageal cancer; 1990–2019.

Regions within middle SDI, higher-middle SDI, low SDI, high SDI, and lower-middle SDI had the lowest to highest age-standardized GDR scores, respectively (1.9, 1.6, 1.2, 1.1, and 0.9) (Fig 2). A map showing the age-standardized GDR score in 1990 and 2019 is presented in Fig 3.

Contains information from OpenStreetMap and OpenStreetMap Foundation, which is made available under the Open Database License, https://www.openstreetmap.org/copyright

## Discussion

Results of the present study, which aimed to explore the quality of care in oesophageal cancer and gender and age disparities in the QCI for oesophageal cancer in the population of patients aged 20 and over, showed that the age-standardized rate per 100,000 for all primary outcome measures including DALYs, death, incidence, prevalence, YLDs, and YLLs decreased globally from 1990 to 2019. However, the age-standardized QCI score for oesophageal cancer showed a growing increase for both sexes. Moreover, there were massive variations in the QCI score both globally and regionally, based on SDI classification, between different age groups, and between different genders. That is, lower SDI regions had lower QCI score than higher SDI regions. Although the lower number of incidence and prevalence of oesophageal cancer were reported in lower SDI regions, ratio of mortality to incidence was higher in those regions. In order to improve the disease management and health outcomes of oesophageal cancer, many high-income countries have developed clinical practice guidelines for diagnosis, treatment and follow-up [33] which could possibly account for higher QCI scores in those countries. The management of esophageal cancer is complicated because, due to the difficulties in identifying patients at high risk and overall poor prognosis of the disease, most cases with esophageal cancer are diagnosed are found incidentally or after symptoms develop and tumors are locally advanced. Evidence show that just one out of eight esophageal cancers is detected at an early stage (T1) [34,35]. Screening for early detection of cancer often allows for more treatment options and is a promising strategy to increase the chance of survival. However, no screening test has been proven to decrease the risk of death from esophageal cancer in people who are at average risk [36]. Therefore, countries like the US, the UK, China, and Europe have started screening high-risk populations for oesophageal cancer [36–38]. Although there is no guarantee that screening high-risk populations would result in decrease in mortality, currently it seems like a most promising strategy towards the management of disease and improvement of QCI even in low-resources regions [35,38].

Considering the several documented challenges to provision of timely and high-quality cancer care in lower income countries and deprived regions [39,40], actions are needed to narrow the gaps between countries of different income and different SDI score. Partnership between authorities and healthcare providers, reallocation of resources, preparing youngers for university educations, establishing more health facilities and cancer centers, and guarantying the populations’ access to a cancer specialist in deprived areas are strategies to tackle the problems in LMICs [39–44]. Strategies such as large-scale commodity purchases in order to reduce the costs of essential inputs, development of technical assistance, and the advancement of cancer research as global initiatives for cancer control in LMICs are recommended as well [42].

QCI score for oesophageal cancer has inconsistently increased globally by increase in age as of age 85. The results showed that there were age disparities among regions considering their classifications by SDI. A pile of evidence have shown socioeconomic inequalities in the cancer care [45–48] and many other studies have highlighted that an area’s socioeconomic status is connected to oesophageal cancer care outcomes in that area [49–51]. For example, patients from rural areas were 22% more likely to die from oesophageal cancer than their counterparts in urban areas [50]. The present study adds to the current literature that such inequalities in oesophageal cancer are not just tailored to individuals’ socioeconomic status but can be seen in forms of global and regional disparities with different age patterns as well. The results showed that elderly patients from lower SDI regions had worse QCI than their peers in higher SDI regions. For example, in higher-middle SDI and high SDI regions, patients from the older age groups had better QCI than the rest.

Elderly patients with esophageal cancer are among the most vulnerable population of individuals with cancer experiencing huge therapeutic challenges due to the aggressive character of the disease [52]. As a result, special attention need to be paid on these groups of patients. However, regions with lower SDI have failed to prepare older patients with QC as equal as the younger groups receive. The little available evidence suggests that access barriers and quality deficiencies in cancer care are determinants of provider delay in LMICs. To our knowledge, research on deficiencies in QC for elderly people with cancer in LMICs are limited, while it is the most required to strengthen health systems to tackle the age disparities in the QC by designing the cost-effective public policies [53]. Evidence on delay in diagnosis and treatment of cancer showed that aged people may experience a large delay in both aspects of the care due to financial difficulties, illiteracy, lack of appropriate health insurance, negative attitude to diagnosis and treatment process, and having no hope or motivation to live longer [53]. Since in the present study the QCI has almost been derived from outcome measures, we suggest that it is very possible to attribute the lower QC in aged population in lower SDI regions to the aforementioned factors as well as to several comorbidities people in older age have.

Our study showed that there globally have been gender disparities in QC among patients with oesophageal cancer through the years 1990 to 2019. This is while it has been known that men are notably at higher risk of this cancer than women are [3]. The pattern of gender disparities showed variations from region to region, however, we could not attribute the disparity to the SDI status of regions. For example, middle SDI regions showed greater GDR than the rest of the regions. Nevertheless, it is evident that female patients take advantage more from the QC than male patients do. A narrative literature review in line with the results of the current study concluded that major disparities in oesophageal cancer care have been omnipresent and no meaningful progress in decreasing them has been achieved [20].

Evidence have cited different possible reasons for gender difference in gastrointestinal and oesophageal cancer care including differences in biological and etiological factors, genetics, hormonal factor, and risk factors [54]. Moreover, differences in risk taking behavior as well as health seeking behavior of female and male may contribute to different health outcomes. Corroborating this theory, evidence show that men are generally more likely than women to take risk, have more harmful alcohol consumption and higher use of illegal psychoactive behavior substances, and get greater involvement in gun accidents [55–57]. There is a study to examine the gender disparity in oesophageal cancer outcomes that shows the disparity is a result of gender differences prognostic factors which is beyond the scope of the current study [58]. Evidence on other types of cancer revealed that male patients’ different health seeking behavior can contribute to delay in diagnosis and treatment and more adverse consequences in comparison to women [59]. For example, men have less propensity to utilize screening and primary healthcare and lower self-health concerns than women [59]. Despite all possible explanations for gender disparity in QC for oesophageal cancer, all mentioned factors could be dependent on socio-cultural factors [60]. The present study revealed a considerable gap in knowledge regarding the reason for gender disparity in oesophageal cancer care while such disparity has a long history since 1990.

### Limitations

Our study has some limitations. First, QCI is restricted to the disease that is being calculated for, here oesophageal cancers. We cannot compare the QCIs of different diseases with each other. Second, while racial/ethnic disparity is one of the fundamental issues of health systems, GBD data are not stratified based on populations’ race/ethnicity. Third, because of the lack of matched data on the risk factors of oesophageal cancers in GBD, we could not adjust the QCI to risk factors. Regardless of all abovementioned limitations, the present study has great strengths that can contribute to better understanding of the QCI and related disparities in oesophageal cancer which could consequently be used in strategic planning and resource allocation. This study is the first report on QCI of oesophageal cancer and showed an acceptable correlation with HAQI.

## Conclusion

Though the age-standardized QCI for oesophageal cancer dramatically increased from 1990 to 2019 for both sexes globally, there were fundamental differences in the QCI between different age groups as well as between males and females. There is a gap in knowledge regarding the reasons for gender and age disparities in the context of oesophageal cancer. To achieve the goal of providing high-quality services equally to people in need in all over the world, health systems need to invest in effective diagnostic services, treatments, facilities, and equipment and to plan for screening and surveillance of high-risk individuals. Countries could contribute to improving the health outcomes of patients with oesophageal cancer through a collective effort in investigations of effective procedures and diagnosis techniques and sharing information with professionals in other countries, particularly those in lower resources regions.

## Abbreviations

DALYs: Disability-Adjusted Life-Years
GBD: Global Burden of Disease
GDR: Gender Disparity Ratio
ICD-10: International Classification of Diseases
LMICs: Low- and Middle-Income Countries
MIR: Mortality to Incidence Ratio
PCA: Principal Component Analysis
QC: Quality of Care
QCI: Quality of Care Index
SDI: Sociodemographic Index
UI: Uncertainty Interval
YLDs: Years Lived with Disabilities
YLLs: Years of Life Lost
